# Identification of an endo-1,4-beta-xylanase of *Ustilago maydis*

**DOI:** 10.1186/1472-6750-13-59

**Published:** 2013-07-26

**Authors:** Elena Geiser, Nick Wierckx, Martin Zimmermann, Lars M Blank

**Affiliations:** 1 iAMB – Institute of Applied Microbiology, ABBt – Aachen Biology and Biotechnology, RWTH Aachen University, Worringerweg 1, Aachen D-52074, Germany

**Keywords:** *Ustilago maydis*, Endo-1,4-beta-xylanase, Xylan, Cell surface display

## Abstract

**Background:**

The utilization of raw biomass components such as cellulose or hemicellulose for the production of valuable chemicals has attracted considerable research interest in recent years. One promising approach is the application of microorganisms that naturally convert biomass constituents into value added chemicals. One of these organisms – *Ustilago maydis* – can grow on xylan, the second most abundant polysaccharide in nature, while at the same time it produces chemicals of biotechnological interest.

**Results:**

In this study, we present the identification of an endo-1,4-beta xylanase responsible for xylan degradation. Xylanase activity of *U*. *maydis* cells was indirectly detected by the quantification of released reducing sugars and could be confirmed by visualizing oligosaccharides as degradation products of xylan by thin layer chromatography. A putative endo-1,4-beta-xylanase, encoded by um06350.1, was identified in the supernatant of xylan-grown cells. To confirm the activity, we displayed the putative xylanase on the surface of the xylanase negative *Saccharomyces cerevisiae* EBY100. The presented enzyme converted xylan to xylotriose, similar to the source organism *U*. *maydis*.

**Conclusions:**

The xylan degradation ability together with its unicellular and yeast-like growth makes *U. maydis* MB215 a promising candidate for the production of valuable chemicals such as itaconic acid or glycolipids from lignocellulosic biomass. Therefore, the characterization of the endo-1,4-beta-xylanase, encoded by um06350.1, is a further step towards the biotechnological application of *U*. *maydis* and its enzymes.

## Background

In recent years, the efficient use of plant biomass, especially non-food lignocellulosic biomass as renewable resource for biotechnological applications has become increasingly important [1]. This lignocellulosic biomass is composed of cellulose, hemicellulose and lignin [2,3]. Xylan is a major component of hemicellulose, which is the second most abundant plant material in nature and constitutes up to 35% of the total dry weight of higher plants [4].

The chemical structure of xylan is complex. It consists of a homopolymeric backbone chain of β-1,4-linked D-xylose units and short side chains including different amounts of α-L-arabinofuranosyl-, O-acetyl-, *p*-coumaroyl-, feruloyl-, D-glucuronopyranosyl- or 4-O-methyl-D-glucuronopyranosyl residues depending on the type of plant [2,3,5]. Due to this complexity, several enzyme classes are involved in the breakdown of xylan. Hemicellulases, such as β-D-galactanases, β-D-mannanases or β-D-xylanases, hydrolyze the 1,4-beta-D-glycosidic linkages anywhere in the xylan chain [2,6,7]. Before extensive degradation of the backbone, other enzymes such as acetylesterases, α-glucuronidases, α-L-arabinofuranosidases, ferulic acid esterases or *p*-coumaric acid esterases remove side chains and substitutes [2,6-8]. Once xylanases have released small xylooligosaccharides, the β-xylosidases cleave the oligomeric fragments, predominantly to xylose [2,6,7]. Additionally, there are many cellulolytic enzymes, which have xylanolytic activity as a secondary function [9]. Synergistic interactions of all these enzymes are required for the efficient degradation of xylan and the exact combination of these enzymes varies from species to species, often with a high degree of redundancy.

Xylan degrading enzymes are produced by a wide variety of aerobic and anaerobic bacteria and fungi, but also algae, protozoa, gastropods and anthropods [2,10-12]. Many of these are saprotrophs, requiring these enzymes for plant degradation and liberation of xylose, a primary carbon source for cell metabolism. Others are plant pathogens requiring hemicellulose degradation for plant cell infection [2,10]. Common well-studied xylan degrading organisms are for instance *Trichoderma* or *Aspergillus* species [12-19]. Most of their xylan degrading enzymes are identified, characterized and also expressed in other xylanase negative organisms such as *Escherichia coli* or *Saccharomyces cerevisiae *[2,12,20-22]. In addition to their natural function, xylanases also have a broad range of industrial biotechnological applications including bio bleaching of wood pulp, treatment of animal feed to increase digestibility, processing of food and hydrolysis of lignocellulosic biomass to sugars, which can subsequently be converted into liquid fuels, solvents and other chemicals [7,12,23,24]. Especially for the latter applications, organisms efficiently breaking down lignocellulosic biomass are required.

The utilization of raw biomass components for the production of valuable chemicals has attracted considerable research interest in recent years. Ideally, these cheap biomass compounds are utilized by microorganisms that (naturally) produce such valuable chemicals. *Ustilago maydis* shows this high biotechnological potential and is known to produce chemicals such as glycolipids, itaconic, malic or succinic acid [25-29]. It is a phytopathogenic fungus and belongs to the group of Basidiomycota. It is a biotrophic parasite that causes smut disease in maize, which is characterized by the formation of tumors, called galls, on aerial plant tissue [30,31]. Couturier et al. identified *Ustilago maydis* as the organism with the best biomass-hydrolysis potential out of 20 sequenced fungi such as *Trichoderma reesei*, *Aspergillus terreus* or *Rhizopus oryzae *[32]. Comparing the genomes and secretomes, *U*. *maydis* showed the widest range of enzymatic activities including 33 hydrolytic enzymes such as polysaccharide hydrolases, polysaccharide lyases and pectin esterases [33]. Nevertheless, the xylanolytic activity of *U*. *maydis* is lower than that of *A*. *niger* or *Fusarium graminearum *[32]. These predictions are in line with Müller et al. proposing that the secretome of *U*. *maydis* contains a complete set of hydrolytic enzymes [33]. In contrast, fungi such as *Magnaporthe grisea* and *F*. *graminearum* have more genes encoding hydrolytic enzymes, 138 and 103 respectively, but the variation is lower [30,32]. However, the biotrophic lifestyle of *U*. *maydis* is geared towards minimizing damage to the host to prevent the release of cell wall fragments, which might trigger plant defense responses [34]. Further, the conditions under which the hydrolytic enzymes are expressed can be very specific [35].

Most of *U*. *maydis*’ enzymes are not at all or not completely characterized so far. Cano-Canchola et al. have described xylanase, pectate lyase, polygalacturonase and cellulase activities in *U*. *maydis*, but these activities were not linked to specific genes or enzymes [35]. In this study, we confirm the ability of *U*. *maydis* MB215 to degrade xylan and identify an endo-1,4-beta-xylanase, encoded by um06350, as one of the major components of xylan degradation.

## Methods

### Strains, media and growth conditions

*Ustilago maydis* strain MB215 (DSM 17144) was used in this work. The fungus was cultivated on YEPS medium consisting of 10 g l^-1^ yeast extract, 20 g l^-1^ D-sucrose, and 20 g l^-1^ peptone for at least 48 h at 28°C and 150 rpm. Physiological experiments were performed in 50 ml minimal medium (pH 5.2) containing 1.6 g l^-1^ NH_4_Cl, 0.5 g l^-1^ KH_2_PO_4_, 0.2 g l^-1^ MgSO_4_, 0.17 g l^-1^ FeSO_4_ and 20 g l^-1^ of varying carbon sources such as xylan from birch wood (Carl Roth GmbH, Germany), glucose and xylose in 500 ml Erlenmeyer flasks at 28°C and 150 rpm. Through the process of autoclaving, xylan was almost completely dissolved. All cultures were inoculated to a starting OD_600_ of 0.5.

*Escherichia coli* DH5α (DSM 6897) was used as a host for DNA manipulation and was grown in lysogeny broth (LB) medium at 37°C and 250 rpm. For plasmid selection, recombinant *E*. *coli* DH5α strains were grown in the presence of 50 mg l^-1^ ampicillin.

The surface display strain *S*. *cerevisiae* EBY100 was cultivated according to the manufacturer’s manual [pYD1 Yeast Display Vector Kit Manual, Invitrogen, Germany].

### Analytical methods

Cell densities were measured by determining the absorption at 600 nm with a Unico spectrophotometer 1201. For dry weight determination 5 ml culture broth was filtered using Macherey-Nagel Paper MN218B (Macherey-Nagel, Germany) and weighed after drying at 110°C for 24 h.

The ammonium concentration in the culture supernatant was measured by a colorimetric method according to Willis using salicylate and nitroprusside [36].

The concentration of reducing sugars was determined by a modified version of the Nelson Somogyi assay [37]. A fresh working solution (12 g l^-1^ K-Na-Tartrate, 24 g l^-1^ Na_2_CO_3_, 16 g l^-1^ NaHCO_3_, 180 g l^-1^ Na_2_SO_4_ and 4 g l^-1^ CuSO_4_ 4•H_2_O) was prepared. 0.5 ml of this solution were added to 0.5 ml sample containing not more than 0.1 g l^-1^ reducing sugars and the mixture was boiled for 15 min. After cooling on ice, 0.5 ml of staining solution (48 g l^-1^ (NH_4_)_6_Mo_7_O_24_ 4•H_2_O, 4.2% (v/v) H_2_SO_4_ and 6 g l^-1^ Na_2_HAsO_4_ 7•H_2_O) were added and mixed for 0.5 min. Reducing sugar concentrations were determined from absorbance at 520 nm. A standard curve was prepared using appropriate amounts of xylose (0–0.1 g l^-1^).

Xylan degradation products were analyzed by thin-layer chromatography (TLC). 5 μl of the culture broth and standards were spotted on a silica gel SIL G-25 TLC plate (20 cm × 20 cm × 0.25 mm, Macherey-Nagel, Germany). 50% (v/v) formate, 33% (v/v) butanol and 17% (v/v) H_2_O were used as running buffer. For staining, the plate was dipped in a mix of 90% (v/v) ethanol and 10% (v/v) of an aqueous 200 g l^-1^ H_2_SO_4_ solution followed by 20 min of heating at 130°C. 20 g l^-1^ xylan, 5 g l^-1^ xylose, 5 g l^-1^ xylobiose and 5 g l^-1^ xylotriose solutions served as standards.

For identification of proteins present in the supernatant of an *U*. *maydis* culture 50 ml culture broth was centrifuged at 7000 rpm for 10 min. The supernatant was lyophilized and resuspended in 5 ml 10 mM Tris–HCl pH 7.5. For protein separation a sodium dodecyl sulfate polyacrylamide gel electrophoresis (SDS-PAGE) on NuPAGE®Novex® 12% Bis-Tris Mini gels in NuPAGE® MOPS SDS Running Buffer was performed according to the manufacturers manual (Invitrogen, Germany). The gel was stained with a 0.23% (w/v) Coomassie Blue R-250 solution for 15 min. Favored slots were cut out and de-stained in 5% (v/v) methanol and 7% (v/v) acetic acid. The de-stained gel slices were washed and equilibrated in 350 μl 30% (v/v) acetonitrile in 0.1 M ammonium hydrogen carbonate for 10 min. Afterwards, the supernatant was discarded and the residual acetonitrile was removed in a Speed Vac vacuum centrifuge. Further steps, such as trypsin-digestion, liquid chromatography peptide fractionation, matrix preparation, MALDI-TOF/TOF mass spectrometry and database search, required for the identification of the expressed proteins, were kindly performed by Benjamin Müller (University Bielefeld, Germany) and are described in Additional file 1.

### Cloning procedures

Standard cloning-related techniques were performed according to Sambrook et al. [38]. As a reference sequence the genomic sequence of *U*. *maydis* 521 (accession number: NW_101210) was used.

A gene replacement construct for disruption of *umxyn11A* (um06350) was constructed by a reverse genetic approach described by Brachmann et al. [39]. An upstream flank was amplified from *U*. *maydis* MB215 genomic DNA with the primers LF-fwd 5’-GTCAATATTCTTGTTAACGATCTCAGCCTCATG-3’ and LF-rev 5’-ACT*GGCCATCTAGGCC*CTTGAATGTTCGAAGAAGAGATCGATGGC-3’ by PCR.

Similarly, the downstream flank was amplified with the primers RF-fwd 5’-AGC*GGCCTGAGTGGCC*ACGTTGAGAGGCCGGATCGGACAGG-3’ and RF-rev 5’- GACAATATTGACCATCAGATTCTTTCAGTCCATGCC-3’. The hygromycin resistance cassette originated from the vector pMF1h [39]. The flanks and the hygromycin resistance cassette were digested with SfiI (indicated by italic sequences) and ligated to obtain the complete replacement construct. This construct was transformed into *U*. *maydis* MB215 protoplasts. Successful homologous integration was tested by colony PCR and single copy integration was verified via Southern blot analysis.

The strain *S. cerevisiae* EBY100/pYD1+*umxyn11A* expressing the putative endo-1,4-beta-xylanase (UmXyn11A) of *U. maydis* MB215 on its cell surface was constructed as follows. The *umxyn11A* gene (um06350) was amplified with primers 5’- AAAAAA*GAATTC*ATGAAGTTTGCCACTGTCCTTGC-3’ and 5’-AAAAAAGA*GCGGCCGC*CAACCAGAGACGGACATCGAGGC-3’ from *U. maydis* MB215 genomic DNA by PCR. The *umxyn11A* gene and the cell surface display vector pYD1 (Invitrogen) were digested with EcoRI and NotI (indicated by italic sequences). Afterwards the fragment was ligated into the vector and transformed into *E. coli* DH5α to obtain the strain *E. coli* DH5α/pYD1+*umxyn11A*. The *umxyn11A* gene was sequenced to confirm the correct sequence and orientation. The transformation of the vector pYD1+ *umxyn11A* in *S. cerevisiae* EBY100 and expression of the UmXyn11A on the cell surface was performed according to the manufacturer’s manual.

## Results and discussion

### Xylan degradation *by Ustilago maydis* MB215

**Figure 1:**
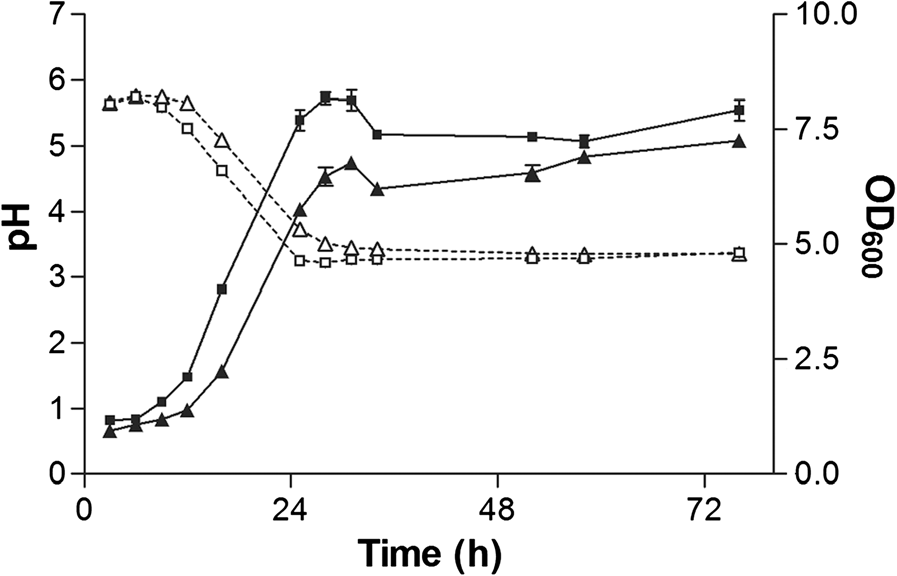
***OD***_***600 ***_***(solid lines, filled symbols) and pH (dashed lines, open symbols) of a *U. maydis *MB215 *(*■,□*) *and *U. maydis *MB215 Δ06350 (▲,Δ) culture in minimal medium containing xylan. ****Error bars indicated deviation from the mean (n=2).*

In order to characterize the xylan degrading abilities of *U. maydis*, strain MB215 was cultivated in minimal medium with xylan as sole carbon source (Figure 1). Growth stopped after 28 h at a maximal OD_600_ of 8.18 ± 0.07, corresponding to 3.82 ± 0.09 g l^-1^ cell dry weight (CDW). The maximal growth rate was 0.10 ± 0.00 h^-1^. Given this low biomass concentration in comparison to the initial substrate of 15 g l^-1^, approximately half of the C-source was likely still present. During the same time the ammonium concentration in the supernatant decreased from 0.58 ± 0.01 g l^-1^ to 0.27 ± 0.01 g l^-1^.

The cessation of growth after 28 h, despite the availability of an N and C source, is probably caused by the pH drop from 5.63 ± 0.01 to 3.37 ± 0.01 (Figure 1). The ammonium consumption of 17 mM accompanied by an equal release of protons lowers the pH by 1.77, which is in line with Figure 1. *U. maydis* is known to secrete small organic acids such as itaconic, 2-hydroxyparaconic, itatartaric, and malic acid [29], but HPLC analysis could not detect any of these in the culture supernatant (data not shown).

**Figure 2:**
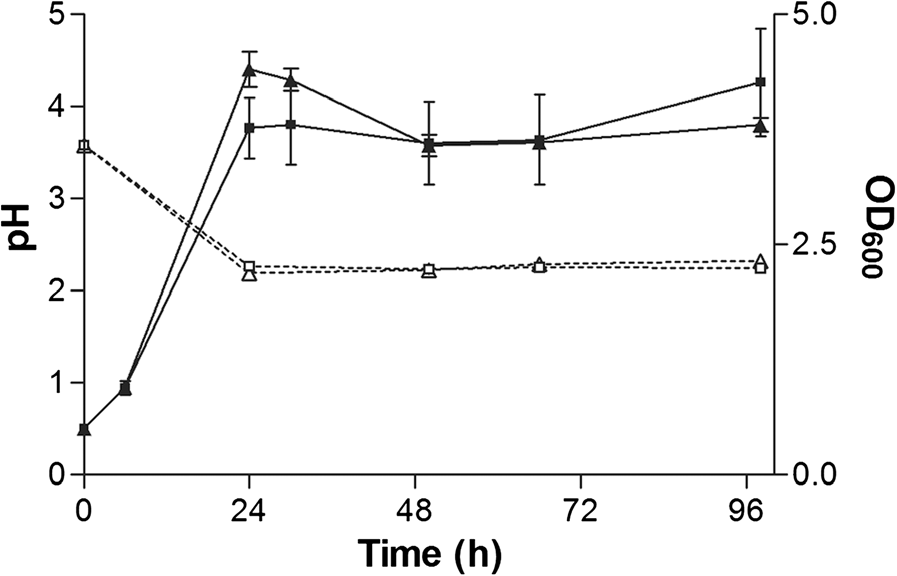
***OD***_***600 ***_***(solid lines, filled symbols) and pH (dashed lines, open symbols) of a *U. maydis *MB215 *(*■,□*) *and *U. maydis *MB215 Δ06350 (▲,Δ) culture in minimal medium containing xylose. ****Error bars indicated deviation from the mean (n=2).*

*U. maydis* species are known to grow at low pH levels down to 2 [40], which was also confirmed by control experiments with xylose as sole carbon source (Figure 2). The cultures on xylose showed a similar growth behavior, pH trend and N consumption with a final cell dry weight of 3.66 ± 0.11 g l^-1^ compared to cultures grown on xylan (Figure 2). Apparently the xylan degrading enzymes had low activity around pH 3 which is in line with other fungal xylanases showing enzymatic activity in a pH range of 3–8 [7].

**Figure 3:**
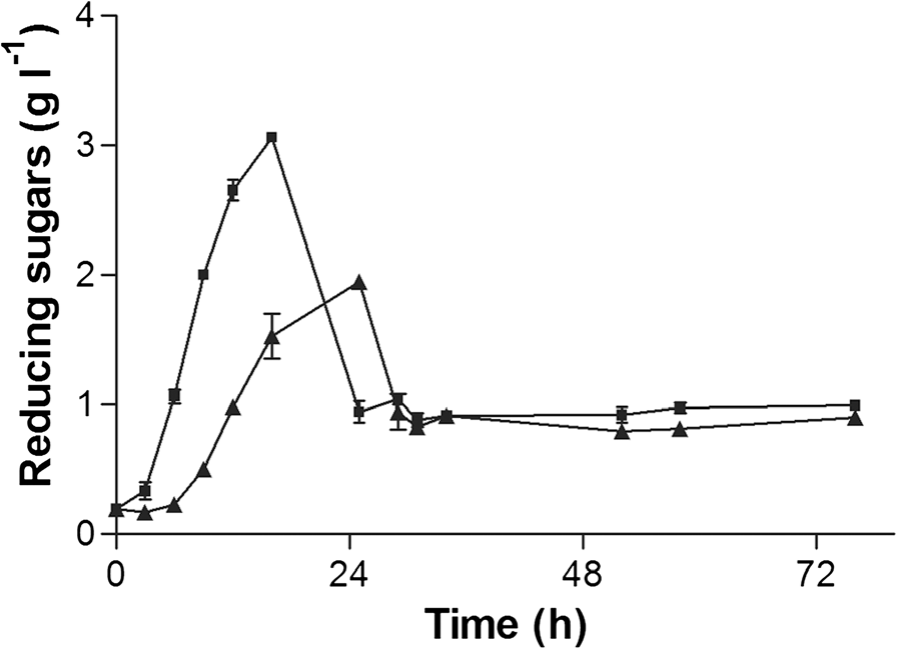
***Concentration of reducing sugars released by *U. maydis *MB215 *(*■*) *and *U. maydis *MB215 Δ06350 (▲) during cultivation in minimal medium containing xylan. ****Error bars indicated deviation from the mean (n=2).*

Xylan degradation was indirectly determined via quantification of released reducing sugars using the Nelson Somogyi method (Figure 3). The maximal reducing sugar concentration was 3.07 ± 0.01 g l^-1^ after 16 h of cultivation. This concentration decreased rapidly after the pH drop at 28 h of cultivation to a stable value of about 0.96 ± 0.03 g l^-1^.

**Figure 4:**
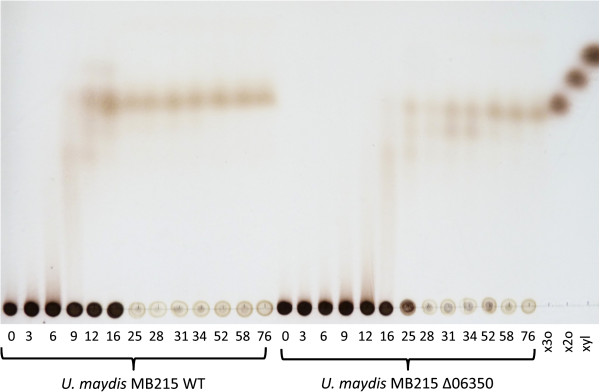
***Analysis of the xylan hydrolysis products in the supernatant of a *U. maydis *MB215 and a *U. maydis MB215 Δ06350 *culture grown on xylan at different time points (in h) by TLC, x3o: 5 g l***^***-1***^***xylotriose; x2o: 5 g l***^***-1***^***xylobiose; xyl: 5 g l***^***-1***^***xylose solution.***

Additionally, xylan degradation was confirmed by visualizing the hydrolysis products via TLC (Figure 4). In the first phase of cultivation different xylo-oligosaccharides accumulated, but after 25 h only xylotriose was left in the supernatant, indicating the presence of a xylanase with endo-acting nature [2,16]. Xylobiose and xylose itself were not found (additionally confirmed by HPLC analysis, data not shown), likely because they were consumed and utilized for biomass formation. These results are in line with the theory of Collins et al. proposing that xylanases are excreted in small amounts into the medium liberating xylo-oligomers, which may be transported into the cell for continuing degradation by beta-xylosidases or intracellular xylanases and induction of further xylanase expression [2]. Intracellular xylanases are known for example in *Bacillus stearothermophilus* or *Prevotella bryantii* as well as intracellular xylosidases in *Klebsiella oxytoca*[23,41,42]. This fast xylo-oligomer uptake and intracellular degradation can also be advantageous in densely populated ecosystems concerning the interspecies competition for nutrients [41,43].

**Figure 5:**
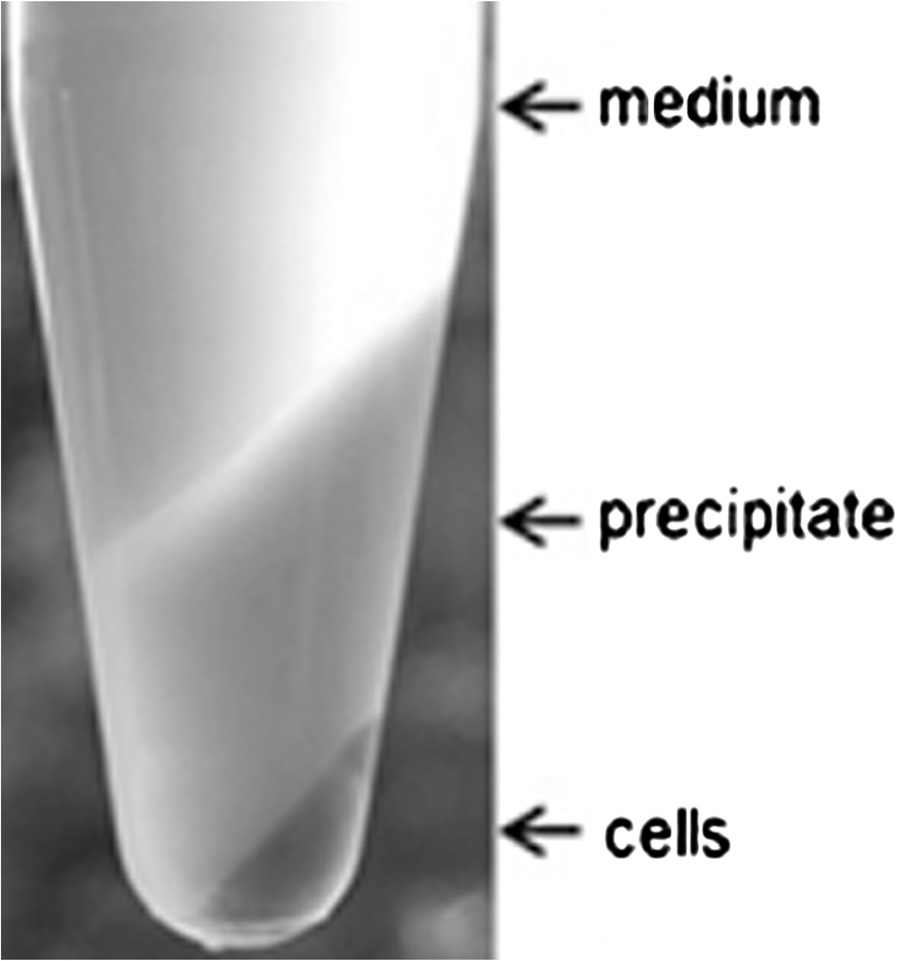
***Centrifuged (6000 rpm, 5 min) culture broth of an *U. maydis *MB 215 culture grown on a minimal medium containing xylan for 25 h.***

Interestingly, after 25 h a gel-like precipitate in high concentrations (up to 4.8 ± 0.64 g l^-1^) was observed (Figure 5). This precipitation coincided with the drop in reducing sugars (Figure 3) and the disappearance of the high molecular weight (HMW) spot at the bottom of the TLC (Figure 4). Control experiments under the same conditions with xylose or glucose instead of xylan did not show this precipitation, nor did it occur in xylan containing medium without inoculation, even when the pH was reduced to 3.2 with DL-malic acid. Thus, the precipitate is likely the result of a modification of the HMW xylan by *U. maydis* MB215. The precipitate was not soluble in water, methanol or ethanol, but it dissolved completely in a 3 M NaOH solution indicating that it contains a carboxylic acid moiety. Possibly, acidic side chains such as glucopyranosyluronic acid formed during the xylan degradation, causing the precipitation in the low pH environment [44].

### Identification of *U. maydis’* xylanases

To find the enzymes responsible for xylan degradation, we searched for probable xylan degrading enzymes in the genome of the reference strain *U. maydis* 521. According to Müller et al. at least 12 open reading frames (ORFs) are present in the secretome of *U. maydis*, whose products could be responsible for the xylan degradation [33]. Four of these enzymes are putative endo-1,4-beta-xylanases (um06350.1, um03411.1, um04422.1 and um04897.1), three alpha-L-arabinofuranosidases (um01829, um03416, um01427), two beta-galactosidases (um02204, um02356) and acetylxylan-esterases (um11763, um04687) and one arabinoxylan arabinfuranohydrolase (um04309) [33].

MALDI-TOF analysis of a *U. maydis* MB215 culture grown on xylan confirmed that the putative endo-1,4-beta-xylanase encoded by um06350.1 was present in the supernatant, together with other biomass degrading enzymes such as arabinoxylan arabinfuranohydrolases, glucanases, glycosyl hydrolases and galactosidases (Table 1). Altogether 14 proteins were found in the supernatant of which 9 have putative biomass degrading activity.

**Table 1:** **Putative proteins in the supernatant of a *U. maydis *MB215 culture grown on xylan detected by MALDI-TOF analysis**

**Putative enzyme activity**	**Gene(s)**	**Score**^**A**^	**M**_**w **_**[kDa]**	**pI**	**Sequence coverage [%]**^**A**^	**Number of peptides**^**A**^	**CAZy family**^**B**^	**Remark**
endo-1,4-beta-xylanase	um06350.1	87/111	23.8	9.41	24.0/18.1	4/3	GH11	xylan degradation
arabinoxylan arabinofurano-hydrolase	um04309.1	149/142	36.3	9.00	21.1/13.6	5/3	GH62	xylan degradation
glycosidase	um02727.1	179/180	25.1	9.27	26.7/26.7	3/3	GH25	D-glucan degradation
endo-1,3(4)-beta-glucanase	um02134.1	331/58	41.5	5.84	17.3/2.3	4/1	GH16	D-glucan degradation
glycosyl hydrolase	um06247.1	65/85	54.0	5.98	4.47/4.4	2/2		(hemi-) /cellulose degradation
chitin deacetylase	um02689.1	-/100	52.4	6.63	-/6.0	-/2	CE4	polysaccharide degradation
lipase B	um01422.1	118/130	35.1	8.81	8.3/8.3	2/2		lipid degradation
aspartic protease	um00064.1	316/338	41.5	5.80	17.1/17.1	4/4		peptide degradation
alpha-galactosidase	um04503.1	153/138	60.5	6.58	8.6/4.7	3/2	GH27	galactomannan degradation
spherulin 4	um06157.1	237/150	35.5	9.67	24.7/14.5	4/3		spherulin-like protein
choline dehydrogenase	um03246.1	222/176	67.9	6.30	8.0/6.5	5/3		glycine, serine and threonine metabolism
uncharacterized protein	um05604.1	88/101	27.0	4.65	6.0/6.0	3/3		unknown
uncharacterized protein	um01894.1	49/48	30.4	5.53	6.5/6.5	2/2		unknown
uncharacterized protein	um00961.1	367/371	31.6	6.19	24.8/20.7	4/3		unknown

^A^: numbers are given for both analyses of a biological replicate.

^B^: CAZy families are signed according to Cantarel et al. [47].

The other probable xylan degrading enzymes were not present in the supernatant, although they were predicted as secreted enzymes containing a signal peptide cleavage site in their amino acid sequences [33,45]. Different regulatory induction mechanisms could be a reason for the absence of these enzymes in the supernatant, since certain external conditions (e.g., substrate variety and concentration, host type and host presence) are required for their expression [35,46].

All proteins identified in Table 1 were also found by Couturier et al. except for one (um06247.1). However, altogether fewer proteins were found in our study, which can be explained by the fact that Couturier et al. used complex media with maize bran as carbon source. This indicates that the induction of different components of the biomass-degrading machinery of *U. maydis* is individually regulated.

In order to characterize the xylan degrading ability of *U. maydis* we concentrated on the identified putative endo-1,4-beta-xylanase. UM06350 is defined as UmXyn11A since the enzyme belongs to the GH11 family [47]. First annotation was done by Couturier et al. [32]. According to the MUMDB Ustilago database the *umxyn11A* gene (um06350.1) has a size of 666 nt and does not contain predicted introns [48].

### Effect of the deletion of um06350 on *Ustilago maydis* MB215

To confirm the xylanase activity of UmXyn11A, we deleted the corresponding ORF and checked the xylan degradation ability of this deletion mutant. Notably, the deletion of the *umxyn11A* gene reduced the growth rate of the mutant *U. maydis* MB215 Δ06350 on minimal medium with xylan to 0.09 ± 0.00 h^-1^ compared to 0.10 ± 0.00 h^-1^ of the wildtype (Figure 1). The deletion mutant reached a final OD_600_ of 6.78 ± 0.00, compared to 8.18 ± 0.07 in the wildtype culture. However, the final CDW of 4.50 ± 0.04 g l^-1^ is similar to the wildtype. The drop of the pH value from initially 5.66 ± 0.00 to 3.36 ± 0.01 was approximately 3 h delayed in comparison to *U. maydis* MB215 (Figure 1). Also the maximal concentration of reducing sugars was lower (1.95 ± 0.03 g l^-1^), and occurred 9 h later than in the wildtype culture (Figure 3). In the end of cultivation the reducing sugar concentration decreased to 0.87 ± 0.03 g l^-1^ similar to the wildtype. Analyzing hydrolytic products via TLC also showed that the release of smaller xylo-oligosaccharides by the deletion mutant was slower than in the wildtype. After 52 h, xylotriose was the only xylo-oligosaccharide detectable in the supernatant. These results indicate that the deletion of the selected *umxyn11A* locus had a negative influence on the rate of xylan degradation of *U. maydis* MB215 Δ06350, although the overall growth on xylan was not affected. Redundant xylan-degrading activity of the other mentioned xylanases was apparently still sufficient to support growth. The deletion mutant growing on xylose as sole carbon source showed the same growth rate and N-consumption as the wildtype indicating no polar effect of the *umxyn11A* deletion (Figure 2). This confirmed the assumption, that the deleted ORF is involved in the xylan degradation and not in growth processes or xylose uptake mechanism.

### Heterologous expression of UmXyn11A on the cell surface of *Saccharomyces cerevisiae*

For further confirmation of the xylanase activity we displayed the UmXyn11A on the cell surface of a xylanase-negative strain, *S. cerevisiae* EBY100. This strain can neither utilize xylan nor xylose nor xylo-oligosaccharides [49]. With the help of the cell surface display vector pYD1, we constructed the strain *S. cerevisiae* EBY100/pYD1+*umxyn11A*, expressing the UmXyn11A attached to the cell surface via disulphide bonds after induction with galactose. The correct expression of UmXyn11A was confirmed by Western analysis (data not shown). The strain *S. cerevisiae* EBY100/pYD1 was used as a negative control.

**Figure 6:**
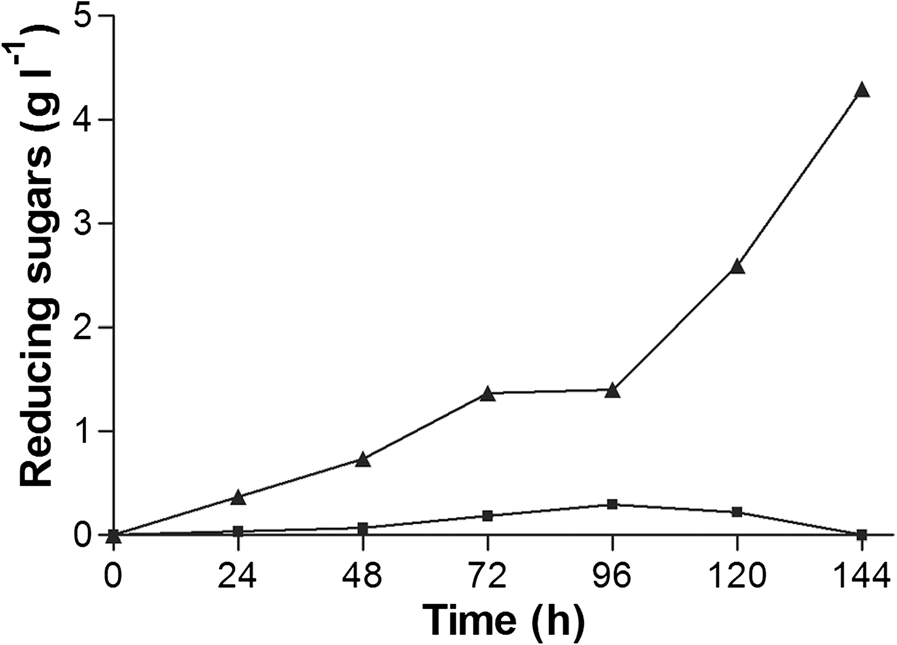
***Concentration of reducing sugars released by *S. cerevisiae *expressing the putative xylanase (▲) and the empty expression vector (negative control, ■) during incubation in xylan solution (n=1).***

The enzyme activity of the displayed UmXyn11A was determined by measuring the increase of released reducing sugars. After 144 h 4.2 g l^-1^ reducing sugars were formed upon incubation of *S. cerevisiae* EBY100/pYD1+*umxyn11A* with 15 g l^-1^ xylan solution by the displayed UmXyn11A, compared to 0.0 g l^-1^ reducing sugars in the negative control not expressing any xylan degrading enzymes (Figure 6). Therefore, the strain *S. cerevisiae* EBY100/pYD1+*umxyn11A* almost completely degraded xylan to xylotriose, which was additionally confirmed by TLC (data not shown).

## Conclusions

This study showed that *U. maydis* MB215 is able to degrade xylan. The recombinant expression of UmXyn11A (um06350.1) supports that it is involved in xylan degradation, and the resulting degradation products are in accordance with the activity of an endo-1,4-beta-xylanase [2,16]. As already suggested by the genome sequence, the deletion mutant shows that *U. maydis* MB215 possesses other genes coding for xylan degrading enzymes. For comparison with other xylanases of well-characterized strains, such as *T. reesei*, further characterization has to be performed concerning the determination of the specific enzyme activity, substrate specificity or pH and temperature optima.

This xylan degradation ability together with its unicellular and yeast-like growth makes *U. maydis* MB215 a promising candidate for the production of valuable chemicals such as itaconic acid or glycolipids from lignocellulosic biomass. Thus, the characterization of this enzyme is a further step towards the biotechnological application of *U. maydis* and its enzymes.

## Competing interests

The authors declare that there are no competing interests.

## Authors’ contributions

LMB and MZ conceived and designed the study. EG performed the experimental work. All authors interpreted experimental data. EG and NW wrote the manuscript. All authors read and approved the submission of the manuscript.

## Supplementary Material

Additional file 1 Methods for trypsin-digestion, liquid chromatography peptide fractionation, matrix preparation, MALDI-TOF/TOF mass spectrometry and database search. Click here for file
