# Supplementary material for: Identification of an endo-1,4-beta-xylanase of Ustilago maydis
Source: BMC Biotechnol. 2013 Jul 26;13:59. doi: 10.1186/1472-6750-13-59 (PMC3737115; doi:10.1186/1472-6750-13-59)
Supplement: Additional file 1 — Methods for trypsin-digestion, liquid chromatography peptide fractionation, matrix preparation, MALDI-TOF/TOF mass spectrometry and database search. [file 1472-6750-13-59-S1.pdf]

Additional file 1:

MALDI-TOF analyses:

The slices were rehydrated in 2 µl trypsin solution (0.01 g l^{-1} trypsin in 3 mM Tris-HCl pH 8.8) for 30 min. After addition of 6 µl 3 mM Tris-HCl pH 8.8 followed by incubation overnight, 12 µl LiChroSolv® (Merck Millipore, Germany) were added. Samples were incubated for 15 min and 150 rpm. Thereafter 10 µl 30 % (v/v) acetonitrile and 0.1 % (v/v) trifluoroacetic acid were added and samples were incubated for 20 min and 150 rpm. New sterile Eppendorf Tubes were washed with 50 % (v/v) acetonitrile and 0.1 % (v/v) trifluoroacetic acid. The proteolytic peptides without gel slices were transferred in the prepared Eppendorf Tubes and separated by nano reversed phase liquid chromatography using an EASY-nLC (Bruker Daltonics, Bremen, Germany). The LC-system was run with solvents water and acetonitrile, respectively, in 0.1 % (v/v) trifluoroacetic acid. Samples were loaded onto a trap column (Biosphere NS-MP-10, 100 µm * 2 cm, C18, 5 µm, 120 Å) for desalting and then separated by an analytical column (Biosphere NS-AC-10, 75 µm * 10 cm, C18, 5 µm, 120 Å). The peptides were eluted from the column applying a linear gradient of increasing acetonitrile content (5 to 95 % (v/v) acetonitrile in 35 minutes, flow rate 300 nl min^{-1}). Elutes were automatically mixed with the matrix and spotted on a MALDI target (AnchorChip 384, Bruker Daltonics) using a Proteineer fc II (Bruker Daltonics). The sample was separated into 96 fractions over a 24 min collection time.

For matrix preparation 36 µl of a matrix stock solution (alpha-Cyano-4-hydroxy cinnamic acid saturated in 90 % (v/v) acetonitrile, 0.1 % (v/v) trifluoroacetic acid) was mixed with 718 µl 0.1 % (v/v) trifluoroacetic acid in acetonitrile, 38 µl 0.1 % (v/v) trifluoroacetic acid and 8 µl 100 mM ammonium dihydrogen phosphate. All mass spectrometric data were acquired using an ultrafleXtreme MALDI-TOF/TOF (Bruker Daltonics). MS spectra (700-4500 m/z) were recorded in positive reflector mode with 4000 shots per MS spectrum. Precursor ions with S/N > 20 were selected for MS/MS fragmentation. Here, 2500 shots were acquired in parent mode and 4000 shots in fragment mode, respectively. MS/MS spectra were processed (smoothing, baseline

subtraction and annotation) using flexAnalysis (Bruker Daltonics). The resulting peak lists were sent to an in-house MASCOT server and searched against NCBI nr Database. The search was performed choosing fungi for the taxonomic category and trypsin as enzyme with one missed cleavage allowed. As variable modification, oxidation (Met) was chosen. The peptide mass tolerance was ± 100 ppm and the fragment mass tolerance ± 0.7 Da. MudPIT scoring and a p-value < 0.05 were used for protein identification. Only proteins with at least 2 identified peptides were accepted.
